# Understanding Resilience Among Undergraduate Nursing Students: Dimensions and Associated Factors

**DOI:** 10.3390/nursrep16090298

**Published:** 2026-08-25

**Authors:** Maria Cristina Queiroz Vaz Pereira, Carolina Miguel da Graça Henriques, Amélia Maria da Fonseca Simões Figueiredo, Ana Lúcia da Silva João

**Affiliations:** 1 Faculdade de Ciências da Saúde e Enfermagem, Universidade Católica Portuguesa, Palma de Cima, 1649-023 Lisbon, Portugal; 2 Life Quality Research Center (CIEQV), Santarém Polytechnic University, Complexo Andaluz, Apartado 279, 2001-964 Santarém, Portugal; alsjoao@uevora.pt; 3 Health Research Network (RISE-Health), Santarém Polytechnic University, Quinta do Mergulhão-Senhora da Guia, 2005-075 Santarém, Portugal; 4 Center for Innovative Care and Health Technology (ciTechCare), Polytechnic University of Leiria, Campus 2–Morro do Lena–Alto do Vieiro Apartado 4137, 2411-901 Leiria, Portugal; carolina.henriques@ipleiria.pt; 5 Health Sciences Research Unit: Nursing, Nursing School of the University of Coimbra, University of Coimbra, Av. Bissaya Barreto, 3046-851 Coimbra, Portugal; 6 Faculty of Health Science and Nursing, Centre for Interdisciplinary Research in Health (CIIS), Universidade Católica Portuguesa, Palma de Cima, 1649-023 Lisbon, Portugal; simoesfigueiredo@ucp.pt; 7 Comprehensive Health Research Center (CHRC), Rua do Instituto Bacteriológico, nº5, 1150-082 Lisboa, Portugal

**Keywords:** resilience, nursing students, nursing education, social support, problem-solving skills

## Abstract

**Background**: Resilience is an important resource in nursing education, supporting students in adapting to demanding academic and clinical environments. However, evidence regarding the factors associated with resilience among nursing students remains inconsistent. This study aimed to examine the factors associated with resilience among undergraduate nursing students, with particular emphasis on gender, age, academic progression, institutional context, and selected sociodemographic characteristics. **Methods**: A cross-sectional quantitative study was conducted with 263 undergraduate nursing students from two higher education institutions in Portugal. Data were collected using the Takviriyanun Resilience Factors Scale, adapted and validated for the Portuguese population. Descriptive and inferential statistical analyses were performed to examine the two dimensions of the scale—Personal and Social Support Skills and Problem-Solving Skills—and their associations with sociodemographic and academic variables. **Results**: Students reported generally favourable levels of resilience-related factors, with higher scores observed in items reflecting personal responsibility and perceived social support. Comparatively lower scores were observed for items related to calmness and patience, confidence, optimism, and perceived social acceptance. A statistically significant gender difference was found in Problem-Solving Skills, with male students showing higher mean ranks than female students (*p* = 0.038); however, the effect size was small (r = 0.13). A moderate positive correlation was observed between Problem-Solving Skills and Personal and Social Support Skills (ρ = 0.394, *p* < 0.001). No statistically significant associations were found between the resilience dimensions and age, year of study, higher education institution, displacement status, place of residence, or religious affiliation. **Conclusions**: Undergraduate nursing students demonstrated generally favourable levels of resilience-related factors. Personal and Social Support Skills and Problem-Solving Skills were positively associated, suggesting a meaningful relationship between interpersonal resources and problem-solving competencies. Although a small gender difference was observed in Problem-Solving Skills, the findings do not indicate substantial gender-related differences in resilience-related factors. Educational strategies aimed at strengthening problem-solving, adaptive coping, emotional self-regulation, and social support may be relevant throughout undergraduate nursing education. Longitudinal and multicentre studies are warranted to further investigate the factors associated with resilience and to evaluate the effectiveness of resilience-focused educational interventions.

## 1. Introduction

Resilience has become a central construct in nursing education because it enables students to adapt successfully to the academic, clinical, and emotional demands of their training while maintaining psychological well-being and engagement with learning [1]. Nursing programmes are characterised by an early integration of clinical practice, exposing students to illness, suffering, ethical dilemmas, and death from the beginning of their education [2]. Simultaneously, students must cope with intensive academic workloads, acquire technical and interpersonal competencies, and progressively develop their professional identity. Experiential learning approaches have been recognised as fundamental in this process, as they promote reflective practice, critical thinking, and adaptive problem-solving in authentic clinical settings [3].

These educational demands make nursing students particularly vulnerable to psychological stress. Feelings of uncertainty, inadequacy, and overload are common, especially during clinical placements or when students perceive limitations in their knowledge and skills [4,5]. Prolonged exposure to these stressors may negatively affect mental health and, in severe cases, increase the risk of adverse psychological outcomes, including suicidal ideation [6]. Consequently, resilience has been recognised as a protective factor that reduces the impact of stress, facilitates adaptive coping, and supports psychological well-being throughout nursing education [1,7]. Several authors therefore advocate the systematic promotion of resilience from the beginning of undergraduate education [8], while others emphasise that nursing curricula should foster emotional and psychological competencies alongside technical knowledge [9].

Resilience is widely recognised as a dynamic and multidimensional process that emerges from the continuous interaction between individual characteristics and environmental resources rather than being a stable personality trait [10]. This socioecological perspective is reflected in Grotberg’s framework [11], which conceptualises resilience through the complementary dimensions of “I have” (external support), “I am” (personal strengths), and “I can” (social and interpersonal competencies). Consistent with this perspective, other authors [12] define resilience as the capacity to adapt positively to adversity by mobilising personal strengths and contextual resources, thereby enabling individuals to overcome developmental challenges successfully.

Current evidence indicates that resilience among nursing students is shaped by a complex interaction of individual, academic, and contextual factors. Social support, coping resources, emotional regulation, academic experiences, and the clinical learning environment have all been identified as important determinants of resilience [1,8,9]. Nevertheless, the influence of sociodemographic and educational characteristics remains inconclusive, with findings varying across populations and educational settings. Resilience research in nursing education is still evolving, underscoring the need for studies that clarify the factors that promote resilient adaptation throughout undergraduate training [13].

Interest in resilience has grown considerably since the COVID-19 pandemic, which emphasised the need to prepare healthcare professionals capable of responding effectively to uncertainty, rapidly changing healthcare environments, and emotionally demanding clinical situations [14]. In this context, resilient healthcare systems depend not only on professionals with sound technical competence but also on individuals equipped with the psychological resources required to adapt to adversity and sustain high-quality care [15]. Consequently, understanding the factors that foster resilience during nursing education has become increasingly relevant for both educational institutions and healthcare systems. According to some authors [16], education for empathy and resilience cannot be independent of one another. Rather, the opposite is emphasised, given that resilience exerts a protective effect that enables the free expression of empathy developed by nursing students throughout their lives. This idea is corroborated by other authors, who state that individual resilience constitutes a variable capable of predicting empathetic behaviour in nursing students [17].

Despite the expanding body of literature, important knowledge gaps remain. Most studies have focused on describing overall resilience levels or examining isolated predictors, resulting in inconsistent evidence regarding the relative contribution of sociodemographic and academic characteristics. Furthermore, few studies have investigated these factors simultaneously within a theoretically grounded resilience framework in Portuguese undergraduate nursing students. As a result, it remains unclear which characteristics are most strongly associated with the different dimensions of resilience and, therefore, which factors may be most amenable to educational intervention.

To address these knowledge gaps, the present study aimed to examine the factors associated with resilience among undergraduate nursing students using the Takviriyanun Resilience Factors Scale [12], a validated multidimensional instrument grounded in Grotberg’s socioecological framework of resilience. Specifically, the study investigated the associations between resilience dimensions and selected sociodemographic and academic characteristics. In addition, it examined the relationship between these resilience dimensions to provide a more comprehensive understanding of the factors associated with resilience in Portuguese undergraduate nursing students. By identifying the characteristics most closely associated with specific dimensions of resilience, the study seeks to generate evidence that can support the development of targeted educational strategies to strengthen resilience, psychological well-being, and academic success throughout undergraduate nursing education.

Based on the existing evidence and the theoretical conceptualisation of resilience as a multidimensional construct, the following hypotheses were formulated:

**H1:**  *Gender is associated with resilience dimensions*.

**H2:**  *Year of study is associated with resilience dimensions*.

**H3:**  *Personal and Social Support Skills are positively associated with Problem-Solving Skills*.

**H4:**  *Sociodemographic characteristics (age, displacement status, place of residence, and religious affiliation) are associated with resilience dimensions*.

## 2. Materials and Methods

### 2.1. Study Design

This study employed a cross-sectional quantitative observational design to examine the factors associated with resilience among undergraduate nursing students. A quantitative approach was considered appropriate because the study aimed to measure resilience-related constructs and investigate their associations with selected sociodemographic and academic characteristics.

The study was observational and non-experimental, with no interventions implemented and data being collected at a single point in time. This design enabled the systematic assessment of resilience using a structured and validated instrument, facilitating the identification of patterns, distributions, and associations among the variables of interest.

In addition, the study examined differences in resilience dimensions according to participants’ characteristics, including gender, age, year of study, institution, displacement status, place of residence, and religious affiliation. By exploring these associations, the study sought to provide a more comprehensive understanding of the factors associated with resilience among undergraduate nursing students.

The study is reported in accordance with the Strengthening the Reporting of Observational Studies in Epidemiology (STROBE) Statement for cross-sectional studies (Supplementary Table S1).

### 2.2. Participants and Sampling

The target population comprised 513 undergraduate nursing students enrolled in the first to fourth years of study at two higher education institutions in central Portugal during the data collection period. The eligibility criteria were enrolment in the undergraduate nursing programme at one of the participating higher education institutions, voluntary participation, and the provision of informed consent. No age restriction was established as an inclusion or exclusion criterion. Age was collected solely as a sociodemographic characteristic and was subsequently examined as a study variable. Most participants were aged between 18 and 25 years; however, two students were aged 30 and 32 years, respectively. As both participants fulfilled all predefined eligibility criteria, they were retained in the study and included in the analyses.

A non-probability convenience sampling strategy was adopted to recruit all eligible students. Given the cross-sectional observational design and the voluntary nature of participation, probability sampling was not feasible. Recruitment was conducted through institutional email invitations sent to all eligible students, ensuring that every member of the target population had the opportunity to participate. Of the 513 eligible students invited to participate, 263 completed the questionnaire and were included in the final sample, corresponding to an overall response rate of 51.3%. Although this approach is widely used in survey-based educational research involving university students [12], it may have introduced selection bias. Specifically, students who were more engaged with their academic programme, more interested in resilience, or more responsive to institutional communications may have been more likely to participate, whereas those with lower academic engagement or limited responsiveness to email invitations may have been underrepresented. Nevertheless, the final sample closely reflected the gender distribution of the target population and exceeded the minimum required sample size. These considerations should be taken into account when interpreting the findings, as the use of convenience sampling and voluntary online participation may limit the generalisability of the results to other undergraduate nursing student populations and educational contexts.

The minimum required sample size was calculated using Cochran’s formula with finite population correction, assuming a 95% confidence level, a 5% margin of error, and a population proportion of 50%, which provides the most conservative estimate when the expected proportion is unknown. Based on the target population of 513 undergraduate nursing students enrolled at the two participating higher education institutions, the minimum required sample size was estimated to be 220 participants. A total of 263 students were included in the study, exceeding this requirement and supporting the adequacy of the sample for the analyses performed.

The gender distribution of the sample closely reflected that of the target population. Male students represented 18.7% of the eligible population and 19.8% (*n* = 52) of the study sample, indicating that they were proportionally represented.

### 2.3. Data Collection

Data were collected between October 2025 and January 2026 through an online questionnaire administered via Google Forms. The survey link was distributed by email to all eligible participants, allowing for voluntary participation. Before completing the questionnaire, participants were informed of the study’s purpose and provided informed consent. Although the available study documentation does not allow confirmation of whether the “Limit to 1 response” setting was enabled in Google Forms, the final dataset was screened for completeness, consistency, and potential duplicate records prior to statistical analysis. No duplicate records were identified in the final dataset. The online format facilitated broad access and ensured efficient data collection while maintaining participants’ anonymity.

The questionnaire included the Takviriyanun Resilience Factors Scale [12], a validated instrument for the Portuguese population. This scale assesses resilience across two dimensions, namely personal and social support skills and problem-solving skills, through a set of structured items rated on a Likert-type scale.

### 2.4. Instrument

Data were collected using the Takviriyanun Resilience Factors Scale, adapted and validated for the Portuguese population [12]. This instrument is designed to assess resilience through a multidimensional perspective, capturing both individual competencies and external support mechanisms.

The scale consists of 21 items organised into two main dimensions: Personal and Social Support Skills and Problem-Solving Skills. The first dimension (items Q1–Q10) evaluates perceptions of social support, the availability of interpersonal resources, and the presence of meaningful relationships. The second dimension (items Q11–Q21) assesses individual capacities for self-regulation, responsibility, planning, and the ability to manage and resolve problems across different contexts.

Responses are recorded using a Likert-type scale with four response options, ranging from 1 (“Strongly disagree”) to 4 (“Strongly agree”), with higher scores indicating higher levels of resilience-related factors. One item (Q3) is negatively worded and requires reverse coding to ensure consistency in the interpretation of the results.

Although the exploratory factor analysis initially yielded a three-factor solution based on the eigenvalue-greater-than-one criterion, the third factor was not retained because the evidence did not support its interpretation as a distinct and interpretable latent dimension. This factor accounted for only 7.9% of the total variance and was characterised by a limited number of salient item loadings, with the associated items showing substantial conceptual overlap with the two dimensions of the original instrument. Consequently, the third factor did not represent a theoretically distinct dimension of resilience, and its retention would have increased model complexity without providing a meaningful improvement in construct interpretability. Furthermore, the eigenvalue-greater-than-one (Kaiser) criterion may overestimate the number of factors when used in isolation. Factor retention was therefore guided by the convergence of statistical evidence, theoretical coherence, conceptual interpretability, and parsimony.

Given that the Takviriyanun Resilience Factors Scale was originally developed and validated as a two-dimensional instrument, comprising Personal and Social Support Skills and Problem-Solving Skills, the theoretically established two-factor structure was retained and subsequently examined through confirmatory factor analysis in an independent subsample. The CFA provided supportive, albeit not unequivocal, evidence for the two-factor structure (RMSEA = 0.055; SRMR = 0.074; CFI = 0.892; TLI = 0.879).

Although the scale has previously been validated for the Portuguese population [12], its psychometric properties were reassessed in the present study because measurement performance may vary across populations and contexts. Given that the current sample comprised undergraduate nursing students from two higher education institutions, it was considered important to verify whether the established factorial structure, construct validity, and internal consistency remained adequate in this specific educational context. This approach is consistent with psychometric recommendations to reassess the performance of measurement instruments when applied to new samples or to different educational and cultural settings.

### 2.5. Ethical Considerations

Authorisation to use the data collection instrument was obtained from its original authors. The Takviriyanun Resilience Factors Scale has been previously validated for the Portuguese population [12], ensuring its suitability for the present study.

Ethical approval was granted by the competent Ethics Committee (Opinion No. CE/IPLEIRIA/61/2025). In addition, formal authorisation to conduct the study was obtained from the participating higher education institutions. All procedures were carried out in accordance with internationally recognised ethical standards for research involving human participants, including the principles set forth in the Declaration of Helsinki.

Participation in the study was voluntary. Before data collection, participants were informed about the study’s objectives, procedures, and their rights as research participants, including the right to withdraw at any time without penalty or consequences. Informed consent was obtained from all participants before completing the questionnaire.

Anonymity and confidentiality were strictly ensured throughout the research process. No personally identifiable information was collected, and all responses were coded and stored securely, with access restricted to the research team.

### 2.6. Data Analysis

Data were analysed using the Statistical Package for the Social Sciences (SPSS), version 30 (IBM Corp., Armonk, NY, USA), and JASP. Prior to analysis, the dataset was screened for completeness, consistency, and potential data entry errors.

The dataset was subsequently examined for missing values. As all questionnaire items were configured as mandatory in the online survey (Google Forms), incomplete questionnaires could not be submitted, and no missing data were identified in the final dataset. Consequently, no missing-data imputation was required, and all statistical analyses were conducted using the complete dataset (*n* = 263).

To assess the construct validity of the Takviriyanun Resilience Factors Scale, the total sample was randomly divided into two independent subsamples. The first subsample (*n* = 131) was used to conduct an Exploratory Factor Analysis (EFA), while the second subsample (*n* = 132) was used to conduct a Confirmatory Factor Analysis (CFA).

To assess the suitability of the data for exploratory factor analysis, the Kaiser–Meyer–Olkin (KMO) measure of sampling adequacy and Bartlett’s test of sphericity were examined. The EFA was conducted in the first independent subsample (*n* = 131). The KMO value was 0.823, indicating good sampling adequacy, while Bartlett’s test of sphericity was statistically significant (χ^2^(210) = 782.6, *p* < 0.001), indicating that the correlation matrix was not an identity matrix and that the variables presented sufficient intercorrelations to support factor extraction. The use of EFA was further supported by the theoretical aim of examining the underlying structure of the Takviriyanun Resilience Factors Scale in undergraduate nursing students, given that measurement properties may vary across populations and contexts.

Exploratory factor analysis was performed using principal component extraction with Varimax rotation. Factors with eigenvalues greater than 1.0 were initially retained. Factor retention was subsequently evaluated by considering the magnitude and pattern of factor loadings, conceptual interpretability, theoretical coherence with the established dimensions of the instrument, and parsimony. Although the initial eigenvalue criterion yielded a three-factor solution, the third factor accounted for only 7.9% of the total variance and did not represent a sufficiently distinct and theoretically interpretable dimension. Consequently, the final factorial structure was based on the two theoretically established dimensions of the instrument and was subsequently examined through confirmatory factor analysis in an independent subsample.

Subsequently, confirmatory factor analysis was conducted using a robust estimator to test the factorial structure identified in the exploratory phase. Model fit was evaluated using multiple goodness-of-fit indices, including the Comparative Fit Index (CFI), Tucker–Lewis Index (TLI), Root Mean Square Error of Approximation (RMSEA), and Standardized Root Mean Square Residual (SRMR). Convergent validity was additionally examined through the Average Variance Extracted (AVE).

Discriminant validity was not formally assessed using established criteria such as the Fornell–Larcker criterion or the heterotrait–monotrait (HTMT) ratio. Nevertheless, the two dimensions showed a statistically significant but moderate association (Spearman’s ρ = 0.394, *p* < 0.001), indicating that they are related while remaining conceptually distinguishable. Given the multidimensional nature of resilience, this association is theoretically consistent with the expectation that personal and social support skills and problem-solving skills represent complementary but distinct dimensions. Nevertheless, the absence of a formal assessment of discriminant validity should be acknowledged as a limitation of the present psychometric evaluation.

Descriptive statistical analyses were conducted to characterise the sample and to summarise participants’ responses to the Takviriyanun Resilience Factors Scale. These included the calculation of absolute and relative frequencies for categorical variables, as well as measures of central tendency (mean) and dispersion (standard deviation) for continuous variables and scale items.

The distribution of the data was assessed using the Kolmogorov–Smirnov test, given the sample size. As the assumption of normality was not met, non-parametric statistical tests were selected for inferential analysis. Specifically, the Mann–Whitney U test was used to compare differences between two independent groups, while the Kruskal–Wallis test was applied for comparisons involving more than two groups. Associations between variables were examined using Spearman’s rank correlation coefficient.

All statistical tests were two-tailed, and a significance level of *p* < 0.05 was adopted. Where applicable, effect sizes were considered to support the interpretation of statistically significant findings.

To ensure the robustness, reliability, and reproducibility of the results, the data analysis process was conducted systematically and independently reviewed by more than one researcher.

## 3. Results

### 3.1. Construct Validity of the Takviriyanun Resilience Factors Scale

Construct validity of the Takviriyanun Resilience Factors Scale was examined using exploratory and confirmatory factor analyses conducted on two independent subsamples. The exploratory factor analysis (EFA), conducted with 131 nursing students, yielded an initial three-factor solution according to the eigenvalue-greater-than-one criterion, accounting for 37.6% of the total variance. Following Varimax rotation, the first, second, and third factors accounted for 16.9%, 12.8%, and 7.9% of the variance, respectively. Although most items showed factor loadings above 0.40, the third factor was interpreted with caution because it was defined by a limited number of salient loadings and its associated items showed substantial conceptual overlap with the dimensions of the original instrument.

The three-factor solution was therefore not retained as the final measurement model. This decision was based on the combined consideration of empirical and theoretical evidence rather than on the eigenvalue criterion alone. In particular, the third factor accounted for a relatively small proportion of variance and did not provide evidence of a conceptually distinct dimension of resilience. Its retention would also have introduced an additional dimension that was not consistent with the theoretically established structure of the Takviriyanun Resilience Factors Scale, which comprises Personal and Social Support Skills and Problem-Solving Skills. Moreover, the eigenvalue-greater-than-one criterion is susceptible to overestimating the number of factors when applied as the sole factor-retention criterion. Consequently, the original two-factor structure was considered the most theoretically coherent and parsimonious representation of the construct and was subsequently tested through CFA in an independent subsample.

The confirmatory factor analysis provided supportive, albeit not unequivocal, evidence for the two-factor structure. The model yielded RMSEA = 0.055 (90% CI = 0.039–0.070) and SRMR = 0.074, whereas CFI = 0.892 and TLI = 0.879 were slightly below the conventional 0.90 criterion. Nevertheless, all factor loadings were statistically significant (*p* < 0.05), indicating significant associations between the observed items and their respective latent dimensions. Taken together, these findings provide support for the theoretically established two-factor structure, particularly considering the acceptable RMSEA and SRMR values, the statistical significance of the factor loadings, and the conceptual coherence and parsimony of the model. However, the results should not be interpreted as unequivocal confirmation of the factorial structure.

Convergent validity was further examined using the Average Variance Extracted (AVE). The AVE values were 0.378 for Personal and Social Support Skills and 0.291 for Problem-Solving Skills, both below the commonly recommended threshold of 0.50. These findings indicate limited evidence of convergent validity in the present sample and suggest that a considerable proportion of item variance may be attributable to measurement error or item-specific variance. Therefore, the two-factor structure should be regarded as providing partial rather than definitive evidence of construct validity. Further psychometric studies in larger and more diverse samples are warranted to assess the stability of the factorial structure and strengthen evidence for convergent validity.

The combination of an interpretable two-factor structure, statistically significant factor loadings, acceptable RMSEA and SRMR values, and previously established theoretical and empirical support for the two-dimensional structure provides a reasonable basis for retaining the original model. Nevertheless, the low AVE values indicate that the psychometric evidence is not uniformly strong and should be considered when interpreting the findings. Further validation studies using larger and more diverse samples are warranted to examine the stability of the factorial structure and to strengthen evidence for convergent validity.

Although the scale has previously been validated for the Portuguese population [12], its psychometric properties were reassessed because measurement performance may vary across samples and contexts. The present study involved undergraduate nursing students from two higher education institutions, representing a specific educational population. Re-examining the factorial structure and reliability in this context therefore provides useful evidence regarding the applicability of the instrument while also allowing potential sample-specific limitations to be identified.

### 3.2. Descriptive Analysis

The sample comprised 263 undergraduate nursing students, predominantly female (80.2%), with a mean age of 20.2 years (SD = 2.05). Most participants were aged 18–25 years and were enrolled in the second (38.0%) or first year (33.8%) of study. Most students attended Institution Y (62.4%), were single (97.3%), and came from urban areas (57.8%) and the central region of Portugal (87.1%). Approximately half of the participants (51.0%) lived away from their usual residence during the academic term, with most of these students travelling weekly to their family home. Regarding religious identification, 51.0% reported affiliation with a religion, predominantly Catholicism (33.8%) and Christianity (15.6%) (Table 1).

Table 2 shows a generally positive pattern of responses across the 21 items of the Takviriyanun Resilience Factors Scale, with “Agree” and “Strongly agree” being the predominant response categories. In the Personal and Social Support Skills dimension, the highest levels of agreement were observed for items related to family and social support, particularly those concerning having trusted family members (72.2% strongly agree) and people who provide support (68.1% strongly agree). For the reverse-coded item Q3, most participants disagreed that they felt limited when seeking support. Similarly, the Problem-Solving Skills dimension showed high levels of agreement, particularly regarding responsibility for one’s behaviour (71.1% strongly agree) and empathy towards others (56.3% strongly agree). Overall, the findings indicate favourable self-perceptions of personal, social, and problem-solving competencies associated with resilience.

Table 3 shows generally favourable levels of resilience-related factors, with most item means exceeding 3 and moderate variability in responses. The highest mean scores were observed for responsibility for one’s behaviour (M = 3.70; SD = 0.47), having trusted family members (M = 3.67; SD = 0.60), and receiving social support (M = 3.67; SD = 0.50). In contrast, the lowest mean scores were found for being calm and patient (M = 2.82; SD = 0.83), feeling that most people like the participant (M = 2.94; SD = 0.62), and being confident, optimistic, and hopeful (M = 2.97; SD = 0.72). Overall, the findings indicate a predominant tendency towards agreement, particularly regarding personal responsibility and social support, although some variability was observed across items.

The Takviriyanun Resilience Factors Scale [12] comprises 21 items organised into two dimensions: Personal and Social Support Skills (Q1–Q10) and Problem-Solving Skills (Q11–Q21).

The Personal and Social Support Skills dimension yielded scores ranging from 1.3 to 4.0, with a mean of 3.4 (SD = 0.41), indicating high levels of perceived social support and personal skills. The Problem-Solving Skills dimension yielded scores ranging from 2.1 to 4.0, with a mean of 3.2 (SD = 0.36), reflecting moderate-to-high levels of perceived problem-solving competencies (Table 4).

Overall, both dimensions showed favourable levels of resilience-related factors, with a slightly higher mean for Personal and Social Support Skills, suggesting a somewhat stronger perception of relational resources and social support than of individual problem-solving skills.

### 3.3. Normality Assessment and Analytical Approach

To test the assumption of normality for the variables under study, the Kolmogorov–Smirnov test was applied, as it is appropriate for samples larger than 50 participants. This test assesses the null hypothesis (H_0_) that the distribution of a given variable in the population follows a normal distribution. The results indicate that, for both dimensions of the Takviriyanun Resilience Factors Scale [12]—Problem-Solving Skills and Personal and Social Support Skills—the significance values were below 0.001 (*p* < 0.05). Consequently, the null hypothesis (H_0_) of normality is rejected.

The assumption of normality was assessed using the Kolmogorov–Smirnov test for both dimensions of the Takviriyanun Resilience Factors Scale. The results indicated that the distributions of Problem-Solving Skills (D = 0.12, *p* < 0.001) and Personal and Social Support Skills (D = 0.09, *p* < 0.001) significantly deviated from normality in the sample (*n* = 263).

Given the violation of the normality assumption, non-parametric statistical tests were considered more appropriate for subsequent analyses, as they do not require normally distributed data.

Table 5 presents the results demonstrating a statistically significant correlation between the two subscales of the Takviriyanun Resilience Factors Scale [12]. Spearman’s correlation coefficient (ρ = 0.394) indicates a positive association of moderate magnitude between the Problem-Solving Skills dimension and the Personal and Social Support Skills dimension. The significance value obtained (*p* < 0.001) is below the conventionally adopted threshold (α = 0.05), leading to the rejection of the null hypothesis (H_0_) and the acceptance of the alternative hypothesis (H_1_). Thus, it is concluded that a statistically significant association exists between the two dimensions of the scale.

The positive direction of the correlation indicates that higher scores on one subscale tend to be associated with higher scores on the other. In practical terms, participants who report higher levels of problem-solving skills also tend to report higher levels of personal and social support skills. This suggests that these dimensions of resilience are interrelated within the study population, reinforcing the notion that relational resources and individual coping competencies may develop in complementary ways.

### 3.4. Resilience Versus Gender

Potential differences in the dimensions of the Takviriyanun Resilience Factors Scale [12] according to gender were examined using the Mann–Whitney U test for independent samples.

A statistically significant difference was observed for Problem-Solving Skills (U = 4468.50; Z = −2.079; *p* = 0.038), with male participants showing a higher mean rank than female participants (151.57 vs. 127.18). However, the effect size was small (r = 0.13), indicating that the magnitude of the difference was limited.

For Personal and Social Support Skills, no statistically significant gender difference was found (U = 4560.50; Z = −1.891; *p* = 0.059), although males also showed a higher mean rank than females (149.80 vs. 127.61). The effect size was small (r = 0.12). Overall, gender was associated with only a small difference in Problem-Solving Skills, with no statistically significant difference observed for Personal and Social Support Skills (Table 6).

### 3.5. Dimensions of Resilience Versus Age

To analyse the association between age and the dimensions of resilience, as well as the relationship between the two subscales of the Takviriyanun Resilience Factors Scale [12], Spearman’s correlation coefficient (ρ) was applied, as this test is appropriate when the assumption of normality is not met (Table 7).

Regarding the relationship between age and the Problem-Solving Skills dimension, a very weak positive correlation was observed (ρ = 0.084), which was not statistically significant (*p* = 0.176). This result indicates that there is no evidence of an association between participants’ age and their level of problem-solving skills in the present sample (Table 7).

Similarly, the relationship between age and the Personal and Social Support Skills dimension revealed an almost non-existent negative correlation (ρ = −0.007), which was also not statistically significant (*p* = 0.915). These findings suggest that age is not meaningfully associated with participants’ perceptions of personal and social support skills (Table 7).

### 3.6. Dimensions of Resilience Versus Year of Study

Differences in the dimensions of the Takviriyanun Resilience Factors Scale [12] according to year of study were examined using the Kruskal–Wallis test.

No statistically significant differences were observed across years of study for either Problem-Solving Skills (H = 7.173; df = 3; *p* = 0.067) or Personal and Social Support Skills (H = 5.084; df = 3; *p* = 0.166). For Problem-Solving Skills, third- and fourth-year students showed higher mean ranks (150.97 and 154.02, respectively) than first- and second-year students (125.18 and 123.33). For Personal and Social Support Skills, the highest mean rank was observed among third-year students (151.70), whereas the lowest was observed among first-year students (122.04). However, these differences were not statistically significant. Overall, the findings do not indicate significant differences in resilience dimensions according to the year of study (Table 8).

### 3.7. Dimensions of Resilience Versus Institution

Potential differences in the dimensions of the Takviriyanun Resilience Factors Scale [12] according to the higher education institution were examined using the Mann–Whitney U test.

No statistically significant differences were observed between institutions for either Problem-Solving Skills (U = 7004.50; Z = −1.871; *p* = 0.061) or Personal and Social Support Skills (U = 7670.00; Z = −0.752; *p* = 0.452). For Problem-Solving Skills, students from Institution Y showed a higher mean rank than those from Institution X (138.79 vs. 120.75), whereas the opposite pattern was observed for Personal and Social Support Skills (136.53 vs. 129.27). However, neither difference reached statistical significance. Overall, the findings indicate comparable levels of resilience across the two institutions (Table 9).

### 3.8. Dimensions of Resilience Versus Being Displaced or Not

Potential differences in the dimensions of the Takviriyanun Resilience Factors Scale [12] according to displaced-student status were examined using the Mann–Whitney U test.

No statistically significant differences were observed between displaced and non-displaced students for either Problem-Solving Skills (U = 8363.50; Z = −0.455; *p* = 0.649) or Personal and Social Support Skills (U = 7774.00; Z = −1.414; *p* = 0.157). Non-displaced students showed a slightly higher mean rank for Problem-Solving Skills (134.17 vs. 129.91), whereas displaced students showed a higher mean rank for Personal and Social Support Skills (138.49 vs. 125.26). However, neither difference reached statistical significance. Overall, the findings indicate comparable levels of resilience across displaced and non-displaced students (Table 10).

### 3.9. Dimensions of Resilience Versus Place of Residence

Potential differences in the dimensions of the Takviriyanun Resilience Factors Scale [12] according to place of residence (rural vs. urban) were examined using the Mann–Whitney U test.

No statistically significant differences were observed between rural and urban students for either Problem-Solving Skills (U = 8422.50; Z = −0.022; *p* = 0.982) or Personal and Social Support Skills (U = 8245.00; Z = −0.315; *p* = 0.753). Mean ranks were virtually identical for Problem-Solving Skills (132.12 vs. 131.91) and only slightly different for Personal and Social Support Skills (133.72 vs. 130.74), with rural students showing marginally higher ranks in both dimensions. Overall, the findings indicate comparable levels of resilience between students from rural and urban areas (Table 11).

### 3.10. Dimensions of Resilience Versus Religion

Potential differences in the dimensions of the Takviriyanun Resilience Factors Scale [12] according to religious affiliation were examined using the Mann–Whitney U test. The inclusion of religious affiliation was supported by the previous literature linking religiosity and resilience [18].

No statistically significant differences were observed between students with and without a religious affiliation for either Problem-Solving Skills (U = 8196.50; Z = −0.727; *p* = 0.467) or Personal and Social Support Skills (U = 8408.50; Z = −0.382; *p* = 0.703). Students without a religious affiliation showed slightly higher mean ranks for both Problem-Solving Skills (135.46 vs. 128.67) and Personal and Social Support Skills (133.82 vs. 130.25), although these differences were small and not statistically significant. Overall, the findings indicate comparable levels of resilience among students with and without a religious affiliation (Table 12).

Overall, the analyses supported two main findings. First, a moderate positive association was identified between Personal and Social Support Skills and Problem-Solving Skills, supporting H3. Second, male students reported slightly higher Problem-Solving Skills than female students, partially supporting H1, although the effect size was small. No statistically significant associations were observed for the remaining sociodemographic variables or for the year of study, providing no support for H2 and H4.

## 4. Discussion

The present study aimed to examine the factors associated with resilience among undergraduate nursing students, with particular emphasis on the influence of gender, academic progression, interpersonal resources, and sociodemographic characteristics. Overall, the findings indicate that participants demonstrated generally favourable levels of resilience, with particularly strong scores in dimensions related to personal responsibility, social support, and problem-solving skills. However, comparatively lower scores were observed in aspects associated with emotional regulation and self-perception, suggesting that these domains may represent areas of greater vulnerability during nursing education.

The comparatively lower scores in emotional regulation and self-perception deserve particular attention. Specifically, students reported lower levels of calmness, patience, confidence, optimism, and perceived social acceptance than in the remaining resilience dimensions. These findings may reflect the demanding nature of undergraduate nursing education, which combines intensive academic requirements with progressive exposure to emotionally complex clinical environments. Throughout their education, nursing students must cope not only with substantial academic workloads but also with the emotional demands of patient care, including exposure to suffering, uncertainty, ethical dilemmas, and increasing professional responsibility. Such experiences may challenge students’ ability to regulate emotions, maintain self-confidence, and preserve a positive self-perception, even when they demonstrate adequate interpersonal support and effective problem-solving abilities.

This interpretation is consistent with previous evidence indicating that nursing students frequently experience elevated levels of stress, anxiety, emotional exhaustion, and psychological burden, particularly during clinical placements [1,14]. Concerns regarding clinical performance, fear of making mistakes, adapting to unfamiliar healthcare environments, and balancing academic and clinical responsibilities have all been identified as factors that may undermine emotional well-being and self-efficacy throughout nursing education. In this context, the relatively lower scores observed in emotional regulation and self-perception may reflect the developmental challenges inherent in professional socialisation rather than deficiencies in resilience itself.

These findings have important implications for nursing education. While the high scores observed in interpersonal support and problem-solving skills suggest the presence of important protective resources, they also highlight the need for educational programmes to place greater emphasis on developing emotional regulation, self-confidence, reflective practice, and adaptive coping strategies. Integrating structured resilience-building interventions, psychological support initiatives, and opportunities for guided reflection throughout both academic and clinical training may strengthen these competencies, ultimately enhancing students’ well-being and preparing them more effectively for the psychological and emotional demands of professional nursing practice.

Regarding H1, which proposed gender differences in resilience dimensions, the findings partially supported this hypothesis. Statistically significant differences were observed only in the Problem-Solving Skills dimension, with male students reporting slightly higher scores than female students. Although the observed gender difference reached statistical significance, its practical relevance appears limited. The small effect size (r = 0.13) suggests that gender accounts for only a minor proportion of the variability in problem-solving skills among nursing students. Consequently, this finding should not be interpreted as evidence of substantial gender-related differences in resilience, but rather as a modest statistical association with limited educational implications. From a practical perspective, these results support the implementation of resilience-promoting interventions for all nursing students, regardless of gender, with particular emphasis on strengthening problem-solving and adaptive coping skills throughout undergraduate education.

This result contrasts with previous research reporting higher resilience levels among female nursing students [19], suggesting that gender differences in resilience may be context-dependent and influenced by cultural, educational, or methodological factors. Differences in self-perception, coping strategies, and response patterns may also contribute to the inconsistent findings reported across studies.

Furthermore, the marked gender imbalance within the present sample, in which more than 80% of participants were female, should be considered when interpreting these results. Although this distribution reflects the demographic profile typically observed in undergraduate nursing programmes, the relatively small number of male participants may have reduced the statistical precision and robustness of the gender comparisons. Consequently, the observed difference in problem-solving skills should be interpreted as a preliminary finding rather than definitive evidence of gender-related differences in resilience, and caution is warranted when extrapolating these results to the broader population of nursing students. The results indicate an absence of significant associations between most sociodemographic variables and resilience dimensions, displaying a trend that diverges from the existing literature. In Portugal, this may be due to the persistence of a more traditional upbringing that encourages problem-solving skills from childhood primarily in males. Conversely, the difficulties faced by women in adulthood may stem from systematic prejudice and discrimination experienced since childhood. What typically affects females in the domestic sphere continues to be replicated at school: they are called upon less frequently, challenged with less rigor, held to lower expectations, and penalized more heavily for their mistakes [20]. This perspective is reinforced by Observador Portugal, which notes that a widespread reality of gender pay gaps and underrepresentation of women in decision-making and leadership roles persists in Portugal [21].

Future research should seek to recruit larger and more gender-balanced samples, ideally across multiple institutions, to determine whether the observed differences can be replicated in more representative populations. Nevertheless, irrespective of gender, the present findings reinforce the importance of systematically promoting problem-solving and adaptive coping skills throughout nursing education, as these competencies are fundamental to resilience and to students’ successful adaptation to academic and clinical challenges.

With regard to H2, which posited that students in more advanced years of study would report higher levels of resilience, the findings did not provide statistical support for this hypothesis. Despite the absence of significant differences, resilience scores exhibited a consistent upward trend across academic years, particularly within the problem-solving dimension. Although this pattern did not reach statistical significance, it may reflect the gradual development of adaptive competencies resulting from cumulative academic and clinical experiences. This interpretation is consistent with previous research suggesting that resilience evolves through exposure to increasingly complex learning and clinical environments. Therefore, while H2 was not formally confirmed, the observed trend highlights the potential contribution of experiential learning and progressive clinical exposure to resilience development among nursing students.

Concerning H3, which proposed a positive association between personal and social support skills and problem-solving skills, the findings provided strong support for this hypothesis. A statistically significant moderate positive correlation was identified between these dimensions, indicating that students who perceived higher levels of interpersonal support also reported stronger problem-solving abilities. This finding reinforces the conceptualisation of resilience as a multidimensional and interactive process in which relational resources play a central role in facilitating adaptive coping. The results are consistent with previous studies highlighting the importance of social support networks [9], peer relationships, and a sense of belonging in fostering adaptation and resilience [22]. Similarly, other authors emphasise the protective role of social support in strengthening resilience [23]. Collectively, these findings suggest that resilience should be understood not merely as an individual characteristic but as a dynamic construct shaped by social and environmental influences.

Regarding H4, which proposed significant associations between sociodemographic variables and resilience dimensions, the findings did not support this hypothesis. No statistically significant associations were identified between resilience and age, displacement status, place of residence, or religious belief. These findings suggest that resilience in undergraduate nursing students may be less strongly associated with relatively stable sociodemographic characteristics than with modifiable psychosocial and educational factors.

The absence of significant associations partially contrasts with previous research. Some authors reported a positive relationship between religiosity and resilience, whereas another study conducted in Portugal among people with chronic pain found that resilience, but not religiosity, acts as a protective factor and a key resource for subjective well-being [18]. while other studies identified spirituality as an important protective resource for nursing students facing academic and clinical challenges [19]. Likewise, Ref. [12] suggested that students living away from home may be more vulnerable to stress and emotional difficulties. However, other studies have similarly found that resilience is more strongly influenced by individual coping strategies, psychological resources, and perceived social support than by demographic characteristics, highlighting the inconsistent evidence regarding the role of sociodemographic factors.

Several factors may account for these findings. The relatively homogeneous age distribution of undergraduate nursing students may have reduced the variability needed to detect age-related differences. Furthermore, religious belief was assessed only as a broad sociodemographic characteristic and did not capture dimensions such as spirituality, frequency of religious practice, or strength of religious commitment, which appear to be more closely associated with resilience. In addition, the shared educational environment may have attenuated individual differences by providing similar learning experiences, peer support, and opportunities for personal and professional development.

Taken together, these findings suggest that resilience among nursing students is likely to be shaped by a complex interaction of personal, social, and educational factors rather than by demographic characteristics alone. Although H2 and H4 were not supported, this pattern should not be interpreted as indicating that these variables are irrelevant to resilience. Instead, it reinforces the multidimensional nature of resilience and suggests that factors not directly assessed in the present study—such as the quality of the clinical learning environment, academic stress, psychological well-being, coping strategies, and perceived social support—may play a more prominent role. Future multicentre longitudinal studies incorporating these variables are warranted to further clarify the determinants of resilience during nursing education.

Overall, the findings indicate that nursing students demonstrated favourable levels of resilience, particularly in the domain of personal and social support, while comparatively lower scores in problem-solving skills suggest opportunities for targeted educational interventions. The significant positive association between the two resilience dimensions further supports the view that strengthening interpersonal support may also enhance students’ adaptive coping capacities. Collectively, these findings reinforce the conceptualisation of resilience as a dynamic process that develops through the interaction between individual competencies and educational environments.

From a psychometric perspective, the present study also provides additional evidence regarding the factorial structure of the Takviriyanun Resilience Factors Scale in Portuguese undergraduate nursing students. Although the exploratory factor analysis initially suggested a three-factor solution based on the Kaiser criterion, a more comprehensive evaluation indicated that the additional factor lacked conceptual coherence, explained only a small proportion of variance, and did not represent a theoretically distinct dimension of resilience. This finding illustrates the recognised limitations of relying exclusively on statistical retention criteria, particularly the eigenvalue-greater-than-one rule, which has been shown to overestimate the number of factors in multidimensional psychological instruments.

Rather than adopting the exploratory solution uncritically, the factorial structure was interpreted in light of the theoretical framework underpinning the instrument and subsequently tested through confirmatory factor analysis in an independent subsample. The CFA supported the original two-factor model, demonstrating acceptable model fit and confirming that this structure provides a more parsimonious, conceptually coherent, and theoretically meaningful representation of resilience in this population. These findings emphasise that decisions regarding factor retention should integrate statistical evidence with theoretical plausibility and construct interpretability. They also support current psychometric recommendations advocating the re-evaluation of measurement instruments across different samples and educational contexts, recognising that psychometric properties are sample-dependent rather than fixed characteristics of an instrument.

### 4.1. Implications for Educational Practice

The findings of this study have several important implications for nursing education. First, given the significant association identified between personal and social support skills and problem-solving skills (H3), educational institutions should prioritise the creation of supportive learning environments that foster peer interaction, mentorship, and collaborative learning. The implementation of structured peer-support initiatives and mentoring programmes may strengthen students’ interpersonal resources while simultaneously enhancing their capacity to cope with academic and clinical challenges.

Second, the partial support for H1, combined with the comparatively lower scores observed in the problem-solving dimension, highlights the need to explicitly incorporate the development of problem-solving and adaptive coping competencies into nursing curricula. Educational strategies such as simulation-based learning, problem-based learning, and structured reflective practice may provide valuable opportunities for students to enhance clinical decision-making, critical thinking, and adaptive responses to complex and unpredictable healthcare situations.

Third, although H2 was not statistically supported, the progressive increase in resilience scores across academic years suggests that cumulative educational and clinical experiences may contribute to resilience development. This finding underscores the importance of providing early, continuous, and progressively challenging clinical experiences throughout nursing education. Such experiences should be accompanied by appropriate supervision, constructive feedback, and emotional support to facilitate students’ adaptation to professional demands and strengthen their resilience over time.

Finally, the absence of significant associations between resilience and most sociodemographic variables suggests that resilience-building initiatives should be implemented across the entire student population rather than being targeted exclusively at specific demographic groups. Institution-wide strategies aimed at promoting psychological well-being, emotional competence, supportive relationships, and a positive learning climate may therefore play a critical role in enhancing students’ resilience, academic success, and long-term professional sustainability.

### 4.2. Implications for Policy and Institutional Strategies

From a policy perspective, the findings support the integration of resilience-building strategies as a fundamental component of nursing education. Rather than being conceptualised solely as an individual attribute, resilience should be embedded within institutional policies and educational frameworks designed to promote student well-being, academic achievement, and professional development.

Higher education institutions should ensure the availability of accessible psychological support services, academic counselling, and structured mentoring programmes to facilitate students’ adaptation to the academic and clinical demands of nursing education. Furthermore, the absence of significant associations between resilience and sociodemographic variables (H4) suggests that institutional interventions may play an important role in fostering equity, ensuring that all students, irrespective of their personal background, have comparable opportunities to develop resilience and adaptive coping capacities.

At a broader level, the incorporation of resilience-related competencies into nursing education standards, curriculum guidelines, and accreditation frameworks may contribute to the adoption of more systematic and consistent approaches across institutions. Such measures would not only support students’ psychological well-being and academic persistence but also contribute to the preparation of a resilient nursing workforce capable of responding effectively to the increasingly complex challenges faced by contemporary healthcare systems.

### 4.3. Limitations

Several limitations should be considered when interpreting the findings of this study.

First, the cross-sectional design limits the interpretation of the observed associations. Because all variables were assessed at a single point in time, temporal precedence cannot be established and causal relationships cannot be inferred. In particular, although a positive association was observed between Personal and Social Support Skills and Problem-Solving Skills, the cross-sectional design does not allow the determination of whether stronger interpersonal support contributes to the development of problem-solving skills, whether students with better coping and problem-solving abilities are more likely to recognise and mobilise social support, or whether both are influenced by other unmeasured factors. Similarly, the study cannot capture changes in resilience throughout undergraduate nursing education or determine whether the observed characteristics predict subsequent resilience. Longitudinal studies are therefore needed to examine temporal relationships and changes in resilience across the different stages of nursing education.

Second, the non-probability convenience sampling strategy represents an important limitation. Participants were recruited voluntarily from only two higher education institutions in central Portugal, despite all eligible students being invited to participate. This recruitment approach may have introduced selection and self-selection bias, as students who were more engaged with their academic programme, more interested in resilience, or more responsive to institutional communications may have been more likely to participate. Conversely, students with lower academic engagement or limited responsiveness to email invitations may have been underrepresented. Although the final sample exceeded the minimum required sample size and its gender distribution closely reflected that of the target population, an adequate sample size does not eliminate the potential for selection bias associated with non-probability sampling. Consequently, the sample should not be regarded as fully representative of Portuguese undergraduate nursing students.

Third, the use of self-report measures may have introduced response and measurement biases. Although the Takviriyanun Resilience Factors Scale is a validated instrument for the Portuguese population, participants’ responses may have been influenced by social desirability, particularly because several items address socially valued characteristics such as responsibility, empathy, self-control, and interpersonal functioning. Participants may therefore have tended to provide more favourable responses, potentially resulting in an overestimation of perceived resilience. In addition, self-report measures capture participants’ perceptions of their own characteristics rather than objective or independently observed behaviours. Future studies could strengthen the assessment of resilience by combining self-report instruments with other sources of information, such as behavioural measures, academic indicators, qualitative approaches, or reports from relevant third parties, where appropriate.

Fourth, the generalisability of the findings is limited by the characteristics and setting of the sample. The study was conducted in only two higher education institutions located in central Portugal and relied on voluntary participation through a convenience sampling strategy. Differences in curricula, institutional resources, academic demands, clinical placements, student characteristics, and sociocultural contexts may influence resilience and its associated factors. Therefore, the findings should be interpreted as reflecting the participating students and should not be assumed to apply directly to all undergraduate nursing students in Portugal or to nursing students in other countries and educational contexts. Multicentre studies involving institutions from different Portuguese regions and, where possible, international settings, together with probability-based sampling strategies, would improve the representativeness and external validity of future evidence.

An additional limitation concerns the psychometric properties of the measurement instrument. Although the Takviriyanun Resilience Factors Scale demonstrated satisfactory internal consistency and an acceptable factorial structure in the present study, the Average Variance Extracted (AVE) values for both dimensions were below the recommended threshold of 0.50, indicating limited evidence of convergent validity. Consequently, the latent constructs may not have captured the intended variance as strongly as recommended, and the observed associations should be interpreted with appropriate caution. Further psychometric evaluation of the scale in larger and more diverse samples is warranted.

Furthermore, resilience is a multidimensional construct influenced by a broad range of individual, psychological, academic, and contextual factors. Although this study examined several sociodemographic and educational characteristics, potentially relevant variables such as academic performance, psychological well-being, perceived stress, coping strategies, personality traits, previous clinical experience, and characteristics of the clinical learning environment were not assessed. Their omission may have resulted in residual confounding and may partly explain the absence of statistically significant associations in some analyses.

Finally, the relatively small number of male participants may have reduced the statistical power of gender comparisons. Although the gender distribution of the sample broadly reflected that of the target population, the statistically significant difference identified for Problem-Solving Skills should therefore be interpreted cautiously, particularly given its small effect size.

Taken together, these limitations indicate that the findings should be interpreted primarily as evidence of associations within the participating sample rather than as causal or broadly generalisable estimates. Future research should consider longitudinal and multicentre designs, probability-based sampling, more diverse populations, and multimethod approaches to the assessment of resilience. Such approaches would help establish temporal relationships, reduce the risk of selection and self-report biases, improve external validity, and provide a more robust understanding of resilience among undergraduate nursing students.

## 5. Conclusions

Within the present sample, undergraduate nursing students demonstrated generally favourable levels of resilience, with Personal and Social Support Skills and Problem-Solving Skills emerging as related dimensions. The observed association between these dimensions is consistent with a multidimensional conceptualisation of resilience, in which individual resources and social support represent complementary aspects of adaptive capacity. However, given the cross-sectional design, these findings should be interpreted as associations rather than causal relationships.

The findings also suggest that resilience may be relevant to how undergraduate nursing students perceive and manage academic and clinical challenges. From an educational perspective, they provide useful evidence to support continued attention to resilience within nursing education, particularly regarding problem-solving, emotional regulation, and social support. Previous research has highlighted the relevance of resilience-focused educational approaches and academic self-efficacy in preparing nursing students for the complexity and emotional demands of clinical practice [24,25,26,27]. However, the present study does not establish whether such strategies improve resilience, and their effectiveness should therefore be examined through appropriately designed intervention and longitudinal studies.

At the institutional level, the findings highlight the relevance of considering student well-being and resilience within educational support structures and nursing curricula. Such approaches may provide a framework for supporting students throughout their academic and clinical experiences, although their specific effects on resilience, academic outcomes, or professional preparedness require further empirical evaluation.

The interpretation and generalisability of these findings should be considered in light of the study limitations. Participants were recruited from only two higher education institutions using a non-probability sampling strategy, and therefore the findings should not be assumed to represent the broader population of undergraduate nursing students. In addition, reliance on self-reported measures may have introduced response or social desirability bias. The psychometric findings also indicate areas requiring further investigation, including the relatively modest variance explained in the exploratory analysis, CFI and TLI values that were below conventional thresholds in the confirmatory analysis, limited evidence of convergent validity based on AVE, and the absence of a formal assessment of discriminant validity.

Future research should therefore employ longitudinal and multicentre designs, preferably involving larger and more diverse samples and, where feasible, probability-based sampling. Further studies should also examine variables such as academic performance, mental health, clinical placement characteristics, and coping strategies to provide a more comprehensive understanding of resilience among nursing students. Additional psychometric studies are warranted to further evaluate the factorial structure, convergent and discriminant validity, and stability of the instrument across different educational, institutional, and cultural contexts.

Overall, the present findings provide evidence of generally favourable resilience levels and a positive association between Personal and Social Support Skills and Problem-Solving Skills among the participating undergraduate nursing students. These findings contribute to the understanding of resilience in nursing education while highlighting the need for further research to establish the stability, external validity, and broader applicability of these observations.

## Figures and Tables

**Table 1:** Sociodemographic characteristics of the sample.

Variable	Category	*n*	%
Gender	Male	52	19.8
	Female	211	80.2
Age	18	44	16.7
	19	65	24.7
	20	67	25.5
	21	38	14.4
	22	19	7.2
	23	7	2.7
	24	8	3.0
	25	13	4.9
	30	1	0.4
	32	1	0.4
Year of course	1	89	33.8
	2	100	38.0
	3	51	19.4
	4	23	8.7
Institution higher education	X	99	37.6
	Y	164	62.4
Marital status	Single	256	97.3
	Married	4	1.5
	Cohabiting	2	0.8
	Divorced	1	0.4
Displaced	No	129	49.0
	Yes	134	51.0
Frequency of visits to usual place of residence (if displaced)	Every week	104	39.5
	Every fortnight	17	6.5
	Once a month	8	3.0
	During the holidays	16	6.1
Living together during term time	Parents	135	51.3
	Friend(s)	39	14.8
	Alone	60	22.8
	Another relative	16	6.1
	Spouse	10	3.8
	Grandparents	3	1.1
Place of residence	Rural	111	42.2
	Urban	152	57.8
Region of origin	Centre	229	87.1
	South	12	4.6
	Islands	13	4.9
	North	6	2.3
	Cape Verde	1	0.4
	Brazil	1	0.4
	São Tomé	1	0.4
Identification with a religion	No	129	49.0
	Yes	134	51.0
Religion	Catholic	89	33.8
	Christianity	41	15.6
	Spirituality	2	0.8

**Table 2:** Distribution of responses to the resilience scale items.

Items from the Takviriyanun Resilience Factors Scale [12]	Strongly Disagree		Disagree		Agree		Strongly Agree	
	*n*	%	*n*	%	*n*	%	*n*	%
Q1 I have people in my family whom I can trust	4	1.5	6	2.3	63	24.0	190	72.2
Q2 I have people outside my family whom I can trust	2	0.8	11	4.2	91	34.6	159	60.5
Q3 I feel restricted when seeking support (reversed)	50	19.0	145	55.1	59	22.4	9	3.4
Q4 I have people who serve as role models for me	8	3.0	17	6.5	120	45.6	118	44.9
Q5 I have people who encourage me to be independent	2	0.8	4	1.5	103	39.2	154	58.6
Q6 I have people who support me	1	0.4	1	0.4	82	31.2	179	68.1
Q7 I have resources available that I can rely on	4	1.5	15	5.7	138	52.5	106	40.3
Q8 I have a stable family and community	9	3.4	25	9.5	127	48.3	102	38.8
Q9 I have people who recognise when I do the right thing	2	0.8	6	2.3	130	49.4	125	47.5
Q10 I feel that most people like me	4	1.5	47	17.9	172	65.4	40	15.2
Q11 I empathise with other people’s feelings	0	0.0	0	0.0	115	43.7	148	56.3
Q12 I respect myself and others	0	0.0	1	0.4	113	43.0	149	56.7
Q13 I am responsible for my behaviour	0	0.0	2	0.8	74	28.1	187	71.1
Q14 I am confident, optimistic and hopeful	4	1.5	59	22.4	140	53.2	60	22.8
Q15 I am generally calm and patient	14	5.3	75	28.5	118	44.9	56	21.3
Q16 I am someone who plans things well	4	1.5	43	16.3	153	58.2	63	24.0
Q17 I prepare myself to deal with anything that might interfere with achieving my goals	1	0.4	40	15.2	168	63.9	54	20.5
Q18 I don’t give up on a task until I’ve finished it	1	0.4	50	19.0	146	55.5	66	25.1
Q19 I solve problems in a variety of contexts	0	0.0	12	4.6	192	73.0	59	22.4
Q20 I manage and control my behaviour	0	0.0	22	8.4	164	62.4	77	29.3
Q21 I view events with a sense of humour	6	2.3	44	16.7	142	54.0	71	27.0

**Table 3:** Descriptive statistics of the resilience scale items.

Items of the Takviriyanun Resilience Factors Scale [12]	Min	Max	Mean	Standard Deviation
Q1 I have people in my family whom I can trust	1	4	3.67	0.60
Q2 I have people outside my family whom I can trust	1	4	3.55	0.62
Q3 I feel restricted when seeking support (reversed)	1	4	2.10	0.74
Q4 I have people who serve as role models for me	1	4	3.32	0.73
Q5 I have people who encourage me to be independent	1	4	3.56	0.57
Q6 I have people who support me	1	4	3.67	0.50
Q7 I have resources available that I can rely on	1	4	3.32	0.65
Q8 I have a stable family and community	1	4	3.22	0.76
Q9 I have people who recognise when I do the right thing	1	4	3.44	0.58
Q10 I feel that most people like me	1	4	2.94	0.62
Q11 I empathise with other people’s feelings	3	4	3.56	0.50
Q12 I respect myself and others	2	4	3.56	0.51
Q13 I am responsible for my behaviour	2	4	3.70	0.47
Q14 I am confident, optimistic and hopeful	1	4	2.97	0.72
Q15 I am generally calm and patient	1	4	2.82	0.83
Q16 I am someone who plans things well	1	4	3.05	0.68
Q17 I prepare myself to deal with anything that might interfere with achieving my goals	1	4	3.05	0.61
Q18 I don’t give up on a task until I’ve finished it	1	4	3.05	0.68
Q19 I solve problems in a variety of contexts	2	4	3.18	0.49
Q20 I manage and control my behaviour	2	4	3.21	0.58
Q21 I view events with a sense of humour	1	4	3.06	0.73

**Table 4:** Descriptive statistics of the resilience scale dimensions.

Subscale	Scale Items	Minimum	Maximum	Mean	Standard Deviation
Personal and social support skills	Q1 I have people in my family whom I can trust; Q2 I have people outside my family whom I can trust; Q3 I feel restricted when seeking support (reversed); Q4 I have people who serve as role models for me; Q5 I have people who encourage me to be independent; Q6 I have people who support me; Q7 I have resources available that I can rely on; Q8 I have a stable family and community; Q9 I have people who recognise when I do the right thing; Q10 I feel that most people like me	1.30	4.00	3.40	0.41
Problem-solving skills	Q11 I empathise with other people’s feelings; Q12 I respect myself and others; Q13 I am responsible for my behaviour; Q14 I am confident, optimistic and hopeful; Q15 I am generally calm and patient Q16 I am someone who plans things well; Q17 I prepare myself to deal with anything that might interfere with achieving my goals; Q18 I don’t give up on a task until I’ve finished it; Q19 I solve problems in a variety of contexts; Q20 I manage and control my behaviour; Q21 I view events with a sense of humour	2.10	4.00	3.20	0.36

**Table 5:** Correlation between resilience dimensions.

Dimensions of Resilience Scale		Problem-Solving Competencies	Personal Competencies and Social Support
Problem-Solving Skills	r	1.000	0.394
	*p*		<0.001
Personal and Social Support Competencies	r	0.394	1.000
	*p*	<0.001	

**Table 6:** Resilience dimensions by gender.

Dimensions of the Resilience Scale	Gender	*n*	Mean Rank	Mann–Whitney U	Z	*p*
Problem-solving skills	Male	52	151.57	4468.50	−2.079	0.038
	Female	211	127.18			
Personal and Social Support Skills	Male	52	149.80	4560.50	−1.891	0.059
	Female	211	127.61			

**Table 7:** Correlations between age and resilience dimensions.

Dimensions of the Resilience Scale		Age
Problem-Solving Skills	r	0.084
	*p*	0.176
Personal and Social Support Skills	r	−0.007
	*p*	0.915

**Table 8:** Resilience dimensions by year of study.

Dimensions of the Resilience Scale	Year of Study	*n*	Mean Rank	H	gl	*p*
Problem-solving skills	1st	89	125.18	7.173	3	0.067
	2nd	100	123.33			
	3rd	51	150.97			
	4th	23	154.02			
Personal and Social Support Skills	1st	89	122.04	5.084	3	0.166
	2nd	100	131.99			
	3rd	51	151.70			
	4th	23	126.89			

**Table 9:** Resilience dimensions by institution.

Dimensions of the Resilience Scale	Institution	*n*	Mean	U	Z	*p*
Problem-solving skills	X	99	120.75	7004.50	−1.871	0.061
	Y	164	138.79			
Personal and Social Support Skills	X	99	136.53	7670.00	−0.752	0.452
	Y	164	129.27			

**Table 10:** Resilience dimensions by displaced-student status.

Dimensions of the Resilience Scale	Condition	*n*	Mid-Range	U	Z	*p*
Problem-solving skills	Not displaced	129	134.17	8363.50	−0.455	0.649
	Displaced	134	129.91			
Personal and Social Support Skills	Not displaced	129	125.26	7774.00	−1.414	0.157
	Displaced	134	138.49			

**Table 11:** Resilience dimensions by place of residence.

Dimensions of the Resilience Scale	Place of Residence	*n*	Mean	U	Z	*p*
Problem-solving skills	Rural	111	132.12	8422.50	−0.022	0.982
	Urban	152	131.91			
Personal and Social Support Skills	Rural	111	133.72	8245.00	−0.315	0.753
	Urban	152	130.74			

**Table 12:** Resilience dimensions by religious affiliation.

Dimensions of the Resilience Scale	Religion	*n*	Mean	U	Z	*p*
Problem-solving skills	No	129	135.46	8196.50	−0.727	0.467
	Yes	134	128.67			
Personal and Social Support Skills	No	129	133.82	8408.50	−0.382	0.703
	Yes	134	130.25			

## Data Availability

Due to ethical considerations and the protection of participants’ anonymity, the data collected and analysed in this study are not publicly available.

## Supplementary Materials

The following supporting information can be downloaded at https://www.mdpi.com/article/10.3390/nursrep16090298/s1, Table S1: Assessment of Compliance with STROBE Reporting Recommendations.

## Public Involvement Statement

Informed consent was obtained from all students, which was a required condition for participation in the study.

## Guidelines and Standards Statement

In compliance with the recommended guidelines and standards, the study was approved by the ethics committee (Opinion No. CE/IPLEIRIA/61/2025).
