# Supplementary material for: Understanding Resilience Among Undergraduate Nursing Students: Dimensions and Associated Factors
Source: Nurs Rep. 2026 Aug 25;16(9):298. doi: 10.3390/nursrep16090298 (PMC13610314; doi:10.3390/nursrep16090298)
Supplement: Supplementary file 1 [file nursrep-16-00298-s001.zip › nursrep-4461532-supplementary.pdf]

Supplementary Table S1. Assessment of Compliance with STROBE Reporting Recommendations

Section	Item	Recommendation	Reported on page
Title and Abstract	1	Indicate the study design in the title or abstract	p. 1 – The study design is explicitly stated in the Abstract as a "cross-sectional quantitative study".
Background	2	Explain scientific background and rationale	pp. 2–4 – The Introduction presents the scientific background on resilience among nursing students, associated factors, and existing knowledge gaps.
Objectives	3	State specific objectives and hypotheses	p. 4 – The overall objective, specific objectives, and hypotheses H1–H4 are presented.
Study design	4	Present key elements of study design	p. 4 – An observational, quantitative, cross-sectional, and non-experimental study design is described.
Setting	5	Describe setting and dates	pp. 4–5 – Undergraduate nursing students from two higher education institutions in central Portugal; data collection took place between October 2025 and January 2026.
Participants	6	Eligibility criteria and selection	pp. 4–5 – Eligibility criteria, informed consent, and convenience sampling are described.
Variables	7	Define outcomes, exposures, predictors	pp. 4–6 – The resilience dimensions and sociodemographic and academic variables analysed are identified, including gender, age, year of study, institution, displacement status, place of residence, and religious affiliation.
Data sources	8	Measurement methods	pp. 5–6 – An online questionnaire and the Takviriyanun Resilience Factors Scale were used. The two dimensions, 21 items, Likert response scale, and reverse-coded item are described.
Bias	9	Address potential sources of bias	pp. 5 and 23–24 – Selection/self-selection bias associated with convenience sampling and voluntary participation is discussed, as well as potential response and social desirability biases.
Study size	10	Explain sample size	p. 5 – Sample size was calculated using Cochran's formula with a finite population correction, assuming a 95% confidence level, 5% margin of error, and a 50% proportion.
Quantitative variables	11	Explain handling of quantitative variables	pp. 5–6 and 8, 12–14 – Age and resilience dimension scores were treated as quantitative variables. Means, standard deviations, minimums, and maximums were reported and, due to non-normality, non-parametric tests were used.
Statistical methods	12	Statistical methods, missing data, etc.	pp. 7–8 – SPSS/JASP, missing-data procedures, EFA/CFA, the Kolmogorov–Smirnov test, Mann–Whitney U test, Kruskal–Wallis test, and Spearman correlation are described, together with a significance level of $p < 0.05$.

Section	Item	Recommendation	Reported on page
Participants	13	Numbers at each stage	p. 5 – 513 eligible/invited students and 263 complete questionnaires included in the final sample are reported, corresponding to a response rate of 51.3%.
Descriptive data	14	Characteristics of participants	pp. 9–11 – Sociodemographic and academic characteristics are presented, including gender, age, year of study, institution, marital status, displacement status, place of residence, region of origin, and religious affiliation.
Outcome data	15	Report outcome data	pp. 11–14 – Results for the 21 scale items, means, standard deviations, and results for the two resilience dimensions are presented.
Main results	16	Estimates and precision	pp. 14–18 – Correlation coefficients, p-values, test statistics, and effect sizes are reported; the CFA also includes 90% confidence intervals for RMSEA.
Other analyses	17	Subgroup analyses	pp. 15–18 – Analyses are conducted according to gender, age, year of study, institution, displacement status, place of residence, and religious affiliation.
Key results	18	Summarise findings	pp. 18 and 24–25 – The Discussion and Conclusions summarise the main findings, highlighting generally favourable resilience levels, the association between Personal and Social Support Skills and Problem-Solving Skills, and the gender difference in Problem-Solving Skills.
Limitations	19	Discuss limitations	pp. 23–24 – Limitations related to the cross-sectional design, convenience sampling, selection bias, self-report measures, generalisability, psychometric properties, and unmeasured variables are discussed.
Interpretation	20	Interpretation	pp. 18–25 – The findings are interpreted in light of previous literature, the proposed hypotheses, and the multidimensional conceptualisation of resilience.
Generalisability	21	External validity	pp. 23–25 – Limited generalisability is explicitly discussed because the study was conducted in only two institutions in central Portugal and used a non-probability sampling strategy.
Funding	22	Funding statement	p. 26 – The manuscript states: "This research received no external funding."
